# Evaluating the treatment outcomes of repetitive transcranial magnetic stimulation in patients with moderate-to-severe Alzheimer’s disease

**DOI:** 10.3389/fnagi.2022.1070535

**Published:** 2023-01-06

**Authors:** Shouzi Zhang, Lixin Liu, Li Zhang, Li Ma, Haiyan Wu, Xuelin He, Meng Cao, Rui Li

**Affiliations:** ^1^Department of Psychiatry, Beijing Geriatric Hospital, Beijing, China; ^2^CAS Key Laboratory of Mental Health, Institute of Psychology, Beijing, China; ^3^Department of Psychology, University of Chinese Academy of Sciences, Beijing, China

**Keywords:** repetitive transcranial magnetic stimulation, Alzheimer’s disease, functional magnetic resonance imaging, individual difference, intervention

## Abstract

The repetitive transcranial magnetic stimulation (rTMS) shows great potential in the treatment of Alzheimer’s disease (AD). However, its treatment efficacy for AD patients in moderate to severe stage is relatively evaluated. Here, we proposed a randomized, sham-controlled, clinical trial of rTMS among 35 moderate-to-severe AD patients. A high frequency (10 Hz) stimulation of the left dorsal lateral prefrontal cortex (DLPFC), 60-session long treatment lasting for 3 months procedure was adopted in the trial. Each participant completed a battery of neuropsychological tests at baseline and post-treatment for evaluation of the rTMS therapeutic effect. Twelve of them completed baseline resting-state functional magnetic resonance imaging (fMRI) for exploration of the underlying neural contribution to individual difference in treatment outcomes. The result showed that the rTMS treatment significantly improved cognitive performance on the severe impairment battery (SIB), reduced psychiatric symptoms on the neuropsychiatric inventory (NPI), and improved the clinician’s global impression of change (CIBIC-Plus). Furthermore, the result preliminarily proposed resting-state multivariate functional connectivity in the (para) hippocampal region as well as two clusters in the frontal and occipital cortices as a pre-treatment neuroimaging marker for predicting individual differences in treatment outcomes. The finding could brought some enlightenment and reference for the rTMS treatment of moderate and severe AD patients.

## Introduction

Alzheimer’s disease (AD) has become the heaviest burden on the public health system worldwide because of its high morbidity and mortality (Gaugler et al., 2022). Patients with AD suffer from progressive memory loss and decline in executive functions, as well as neuropsychiatric symptoms, which significantly impact the daily living activities and quality of life of patients, and lead to a heavy burden of care (McKhann et al., 2011). Currently available medications are less effective in reducing cognitive and psychobehavioral symptoms and in modifying the progression of the disease (Krantic, 2017). Nonpharmacological interventions such as cognitive training, physical exercise, art-oriented therapy, and brain stimulations for AD have recently been promoted in clinical practice (Krantic, 2017; Sikkes et al., 2021). Among these nonpharmacological interventions, the noninvasive repetitive transcranial magnetic stimulation (rTMS) as a potentially safe and cost-effective treatment has been widely used in treating certain symptoms of various mental illnesses such as depression and insomnia, or neurodegenerative diseases such as AD and Parkinson’s disease (Lefaucheur et al., 2014; Cao et al., 2018).

rTMS releases brief pulses through a coil, generating a time-varying magnetic field pulse, which penetrates the scalp and skull, inducing a secondary current in the brain. Its therapeutic effects may lie in the possibility to promote changes in synaptic plasticity through a number of processes in the brain, including the long-term potentiation (LTP) of excitatory neurotransmission, long-term depression (LTD) of GABAergic synaptic strength, cerebral blood flow, neural functional interactions, and gene expression (Chervyakov et al., 2015). The pathology of AD including the amyloid-β and tau accumulation results in widespread structural and functional brain network disconnections, causing cognitive impairment (Yu et al., 2021). Evidence has indicated that rTMS attenuates synaptic plasticity impairment and neuroinflammation in AD transgenic mice (Li et al., 2021a) and induces LTP-like cortical plasticity in AD patients (Li et al., 2021b), suggesting its important value in the treatment of AD (Teselink et al., 2021). However, the effects of rTMS on cognitive and psychobehavioral symptoms are inconsistent across studies owing to heterogeneity in both experimental trials, such as rTMS frequency and treatment duration, and disease progression, such as the early or late stage of AD. Several meta-analyses have consistently confirmed the effectiveness of using high-frequency stimulation with left dorsolateral prefrontal cortex (DLPFC) as the stimulation target for mild-to-moderate AD (Cheng et al., 2018; Wang et al., 2020). Note that so far, most studies adopted a relatively short duration of treatment sessions, and recent evidence suggests that longer treatment interventions are more effective in improving AD-associated cognitive performance (Lin et al., 2019). For instance, Koch et al. (2022) recently conducted a 24-week period, 32-session precuneus rTMS to slow down the cognitive and functional decline in mild-to-moderate AD. Besides, the efficacy of rTMS in the treatment of moderate and severe AD is less evaluated.

There is often pronounced individual difference in the magnitude of gains from interventions. The individual difference is also a potential contributor to the mixed findings of previously observed rTMS treatment outcomes in AD. The baseline individual difference in brain state may play a role in treatment efficacy, and the identification of a pretreatment neural biomarker could objectively contribute to optimizing the design of personalized treatment options (Francesco and Koch, 2021). Resting-state functional magnetic resonance imaging (fMRI) provides a wide analysis platform to quantify the functional patterns of the brain by calculating the intrinsic functional connectivity (FC) among spontaneous blood-oxygen-level-dependent (BOLD) variations in distributed brain areas (Hojjati et al., 2017). Baseline FC has been demonstrated to predict AD progression and intervention outcomes. For instance, Buckley et al. (2017) found that lower baseline functional connectivity functional networks predicted more rapid decline in preclinical Alzheimer cognitive composite scores over time. The baseline and longitudinal hippocampal functional connectivity is associated with the cognitive changes in progression of MCI and AD (Wang et al., 2011; Dautricourt et al., 2021). Yin et al. (2014) reported that individual differences in the baseline amplitude of fluctuations were correlated with intervention-related changes in behavioral performance. Gallen and D’esposito (2019) further suggested that a more modular network architecture characterizing a more efficient brain at baseline confers cognitive plasticity during interventions. In rTMS of depressive disorders, baseline FC has been successfully used as a predictor of depression alleviation (Whitton et al., 2019). These studies underscore the potentiality of considering functional neuroimaging for personalized diagnostics and therapeutics in diseases (Finn and Constable, 2016).

In this study, to further evaluate the treatment efficacy of rTMS in moderate-to-severe phase of AD, we conducted a randomized, sham-controlled, long course (60 sessions within 3 months), left DLPFC targeted rTMS intervention. A set of cognitive and non-cognitive neuropsychological measurements was used to evaluate the treatment efficacy, and baseline FC obtained from resting-state fMRI was attempted as a biomarker to predict individual difference in treatment outcomes.

## Materials and methods

### Design

A randomized double-blind controlled trial was conducted in the Department of Psychiatry at Beijing Geriatric Hospital (BGH). This study was approved by the BGH Ethics Committee. All enrolled participants were admitted to the department from March to October 2021. After written informed consent was obtained from the patients and their guardians, participants underwent screening and baseline procedures and were block randomized to the rTMS group or sham control group *via* an interactive voice response system. Neither the participants nor the physicians knew whom they had received stimulation therapy.

### Study participants

All the participants enrolled were 60–90 years old patients meeting the National Institute on Aging-Alzheimer’s Association (NIA-AA) AD criteria for the moderate-to-severe phase, with whom Clinical Dementia Rating (CDR) assessment was 2 or 3 (McKhann et al., 2011). The inclusion criteria were eligibility and desire to participate in the study, a reliable informant caregiver, no deficits in hearing or vision, 8th grade education at least, and stable doses of medication for ChEI or memantine for more than 1 month. Exclusion criteria were prominent agitation; use of benzodiazepines or barbiturates up to 2 weeks before screening; history of seizures or diagnosis of epilepsy; contraindication for MRI or rTMS; alcoholism, drug addiction, or severe sleep deprivation; psychiatric disorders other than AD; and severe medical disorders, such as cardiovascular and cerebrovascular diseases or pulmonary infection. All the demographic data, onset time of cognitive impairment and APOE genotype of the subjects were obtained. Overall, 37 subjects were enrolled and block randomized to the rTMS treatment (*n* = 19) or sham control group (*n* = 18).

### rTMS treatment

A Magstim Super Rapid^2^ (Magstim Company Ltd., Whitland, Wales, United Kingdom) with an air-cooled figure-eight coil was used. Each participant received 60 sessions of rTMS treatment within 3 months. Each session lasted 20 min once a day in a course of 20 consecutive days, with an interval of 10 days. The rTMS was administered at 10 Hz for 4 s, with 16 s between the trains. The total stimulation was 2,400 pulses per day with a 100% motor threshold (MT). The left DLPFC was selected as the stimulating target due to its robustness and reliability in treatment effect as demonstrated repeatedly in previous empirical studies (Cheng et al., 2018; Wang et al., 2020).

Cortical MT was defined as the minimum stimulating intensity at which a slight contraction of the contralateral muscle abductor pollicis brevis emerged. The left DLPFC was 5 cm anterior to the hand motor area along the parasagittal line. The coil of the rTMS machine was placed tangentially to the scalp to ensure that the left DLPFC was directly below the center of the coil. The sham stimulation participants received the same treatment as the active intervention participants, except that the coil of the TMS machine was placed parallel to the scalp. The stimulation protocol adhered to the guidelines of the International Federation of Clinical Neurophysiology safety guidelines (Wassermann, 1998).

### Neuropsychological assessment

A battery of cognitive and non-cognitive assessments was performed at baseline and after the end of treatment to evaluate the effects of rTMS. These assessments included cognitive assessment (Mini-Mental State Examination [MMSE] (Folstein et al., 1975); Montreal Cognitive Assessment [MoCA]; (Nasreddine et al., 2005); severe impairment battery [SIB]; (Schmitt et al., 1997)), functional ability and quality of life assessment (activities of daily living [ADL]; Lawton and Brody, 1969), psychiatric assessment (neuropsychiatric inventory [NPI]; Cummings et al., 1994), and comprehensive assessment (Clinician’s Interview-Based Impression of Change plus caregiver input [CIBIC-Plus]; Schneider et al., 1997).

### Image acquisition

Participants who were able to tolerate the MRI process underwent resting state fMRI scanning at baseline and after completing rTMS treatment. A 3-Tesla MRI scanner (Ingenia, PHILIPS, Netherlands) equipped for echo-planar imaging at the BGH MRI Center was used for image acquisition. During the scan, the participants were lying in the supine position with their heads snugly fixed by a belt and foam pads to minimize head motion. Participants were instructed to lie quietly, keep their eyes closed, not think of anything in particular, and not fall asleep throughout the session. For each participant, 160 functional images were obtained using the following parameters: time repetition (TR) = 3,000 ms, time echo (TE) = 35 ms, field of view (FOV) = 240 mm × 240 mm, 40 axial slices, thickness = 4.0 mm, gap = 0 mm, acquisition matrix = 120 × 116, voxel size = 2 × 2 mm, and acquisition times of 10 min and 9 s. In addition, a high-resolution 3-D T1-weighted structural image was acquired for each participant, using the following parameters:180 slices, acquisition matrix = 160 × 211, voxel size = 1 mm × 1 mm × 1 mm, TR = 7.9 ms, TE = 3.5 ms, gap = 0 mm, and acquisition time of 4 min and 58 s.

### Neuropsychological data analysis

Baseline comparisons between the two groups were performed using Wilcoxon test, chi-square test, and *t* tests (*p* < 0.05). The intervention effect of rTMS on behavioral measurements was conducted using a 2 (group, rTMS group vs. control group) × 2 (time, pre-treatment vs. post-treatment) repeated-measures ANOVA (group × time) and post-hoc paired *t*-test (*p* < 0.05). Statistical analyses of behavioral data were conducted using SPSS 21.0 (IBM Corp. in Armonk, NY, United States). The pretest-posttest-control effect size *d_ppc_* for ANOVA (Morris, 2008) and Cohen’s *d* effect size for t-tests (Cohen, 1988) were also calculated.

### Neuroimaging data analysis

#### Preprocessing

Resting-state fMRI data were preprocessed and analyzed using the CONN20b toolbox.^1^ Preprocessing was conducted following the default preprocessing pipeline for volume-based analyses in CONN (Nieto-Castanon, 2020). It includes realignment, slice-timing correction, outlier identification, normalization to Montreal Neurological Institute (MNI) space (3 mm^3^), and smoothing with a Gaussian kernel of 8 mm full-width half-maximum. An anatomical component-based noise correction procedure (aCompCor) was adopted to exclude several confounding effects, including noise components from the white matter and cerebrospinal areas, 24 subject-motion parameters, scrubbing parameters of outlier scans, and the linear signal trend. Subsequently, temporal band-pass filtering (0.01–0.08 Hz) was carried out to reduce the effects of low-frequency drift and physiological high-frequency noise. Additionally, baseline total gray matter percentage of total intracranial volume was calculated to evaluate the gray matter atrophy for each participant by performing the voxel-based morphometry (VBM) processing pipeline for T1-weighted structural images in the Computational Anatomy Toolbox 12 (CAT12).^2^

#### Functional connectivity multivariate pattern analysis (FC-MVPA)

We used data-driven voxel-wise FC-MVPA in CONN20b (Nieto-Castanon, 2020) to explore whether any region whose baseline FC is associated with differences in gains from rTMS. FC-MVPA maps the global multivariate FC patterns between all voxels across all subjects using principal component analysis to create a low-dimensional multivariate representation for each voxel. Pearson’s correlations between baseline FC-MVPA maps and changes in behavioral performance in the rTMS group were examined. Regions significant at a voxel *p* < 0.001 and cluster-size false discovery rate (FDR) corrected *p* < 0.05 were identified as possible neuroimaging markers indicating individual differences in treatment outcomes. To examine whether the identified regions are specific to the rTMS effect, we tested the correlations between these baseline FC and behavioral changes in the control group.

## Results

### Baseline participant characteristics

All patients completed the experimental program and tolerated it well with no reports of adverse effects, except for two patients who withdrew from the trial for severe complications of AD. Finally, 35 subjects were included, with 18 in the rTMS group and 17 in the sham control group. There was no significant difference between the rTMS and sham groups (all *p*s > 0.05) with regard to age, education, sex, average time from AD diagnosis, AD medications, assessment measures, r-TMS motor threshold, or ApoE gene variation. Twelve of them completed the functional MRI scans: six in the TMS group and six in the control group, and no significant difference was found in gray matter atrophy between the two groups at baseline (*p* = 0.19). Table 1 shows the details of the baseline characteristics of participants in the two groups.

**Table 1:** Baseline participant characteristics.

Characteristics	rTMS group	Control group
Total patients, no.	18	17
Age, mean (SD)	84.8 (5.6)	83.4 (4.1)
Male sex, no. (%)	10 (55)	11 (64)
Completed college, no. (%)	8 (44)	9 (52)
Average time from AD diagnosis (y) (SD)	5.4	5.8
Medicated for AD (either ChEI or memantine or both) no. (%)	18 (100%)	17 (100%)
MMSE, mean (SD)	4.7 (3.9)	3.4 (3.7)
MOCA, mean (SD)	3.2 (4.3)	1.8 (2.7)
NPI, mean (SD)	22.4 (5.9)	24.2 (6.1)
CIBIC-plus, mean (SD)	27.2 (6.3)	31.1 (5.7)
ADL, mean (SD)	39.4 (25.7)	42.9 (35.8)
SIB, mean (SD)	30.7 (26.1)	17.7 (22.7)
r-TMS motor threshold, mean (SD)	58.5	59.1
APoE4/4 or ApoE4/3, no. (%)	7 (38.9)	7 (41.2)
*Gray matter percentage, mean (SD)	33.9 (2.6)	27.1 (11.9)

AD, Alzheimer’s disease; MMSE, Mini-Mental State Examination; r-TMS, repetitive transcranial magnetic stimulation; NPI, neuropsychiatric inventory; MoCA, Montreal Cognitive Assessment; CIBIC, Clinician’s Interview-Based Impression of Change; SIB, severe impairment battery; ADL, activities of daily living. None of the differences were statistically significant. *Patients with fMRI data.

### rTMS treatment outcomes

**Figure 1:**
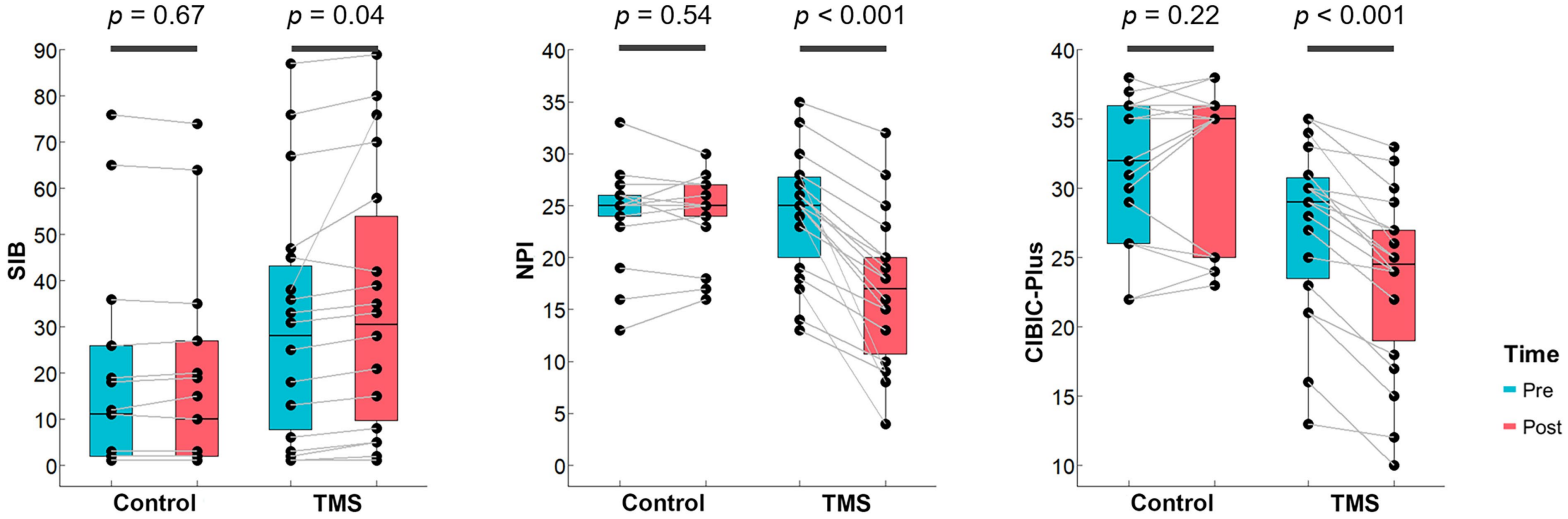
rTMS treatment outcomes on neuropsychological performance. Bar plots show the distributions of scores on SIB, NPI and CIBIC-plus that are with significant Group × Time interactions for both groups. The midline in the box represents the median. Each dot denotes 1 participant.

Using the two-way repeated-measures ANOVA, we found significant interactions between the rTMS treatment group and sham control group in SIB (*F* = 4.18, *p* = 0.049, *dppc* = 0.18), NPI (*F* = 43.20, *p* < 0.001, *dppc* = 1.40), and CIBIC-plus (*F* = 36.0, *p* < 0.001, *dppc* = 0.74). The post-hoc paired *t*-test showed that the SIB score of the rTMS group increased significantly (*p* = 0.04, Cohen’*d* = 0.52), and the NPI (*p* < 0.001, Cohen’*d* = 1.67) and CIBIC-plus (*p* < 0.001, Cohen’*d* = 1.73) scores decreased significantly after the treatment (Figure 1). There was no significant change in SIB, NPI, or CIBIC-plus after sham stimulation in the control group (all *p*s > 0.05). No significant interaction was found in MOCA (*F* = 1.34, *p* = 0.26), MMSE (*F* = 2.56, *p* = 0.12), or ADL (*F* = 0.97, *p* = 0.33).

### Baseline FC-MVPA predicts rTMS treatment efficacy

**Figure 2:**
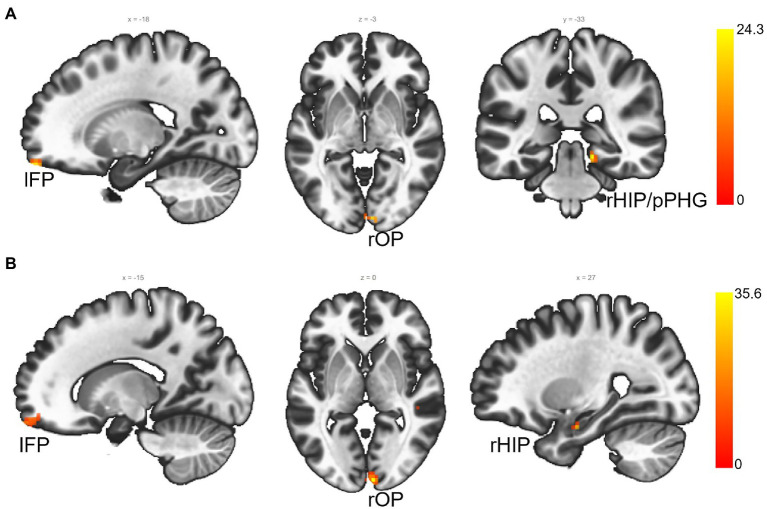
The FC-MVPA that predicts rTMS treatment outcomes at baseline (voxel *p* < 0.001, cluster-size FDR corrected *p* < 0.05). **(A)** ROIs that predict SIB changes from rTMS; **(B)** ROIs that predict CIBIC-Plus changes from rTMS. lFP, left frontal pole; rOP, right occipital pole; right hippocampus/posterior parahippocampal gyrus (rHIP/pPHG); rHIP, right hippocampus.

Before using the baseline FC-MVPA to correlate with treatment outcomes in rTMS group, we first compared the FC-MVPA between two groups, and no significant baseline difference was found (voxel *p* < 0.001, cluster-size FDR corrected *p* < 0.05). Correlation analyses of baseline voxel-wise FC-MVPA maps with behavioral (SIB, NPI, and CIBIC-plus) changes (voxel *p* < 0.001, cluster-size FDR corrected *p* < 0.05, Figure 2) in the rTMS group identified the right hippocampus/posterior parahippocampal gyrus (rHIP/pPHG), left frontal pole (lFP) and right occipital pole (rOP) significantly predicting changes in SIB (rHIP/pPHG: 15, −33, −12; 38 voxels; lFP: −18, 60, −21; 11 voxels; rOP: 6, −90, −3; 10 voxels) and CIBIC-plus (rHIP: 27, −9, −21; 13 voxels; lFP: −15, 66, −18; 29 voxels; rOP: 6, −93, 0; 34 voxels). The FC-MVPA values of above clusters in sham control group were also extracted to correlate with behavioral changes, but no significant correlation was found (all *p*s > 0.05).

## Discussion

By extending previous studies of rTMS in mild-to-moderate AD patients, we used a randomized, sham-controlled trial to demonstrate the positive therapy effect of rTMS in alleviating cognitive and psychobehavioral symptoms for patients with moderate and severe AD. We also demonstrated that baseline multivariate FC of the right hippocampus/posterior parahippocampal gyrus and two clusters in the frontal and occipital cortices predicts individual difference in treatment outcomes.

First, our results confirmed the effectiveness of 10 Hz high-frequency stimulation over the left DLPFC in moderate-to-severe AD patients. The DLPFC plasticity is associated with various cognitive performance in AD, and thus potentially becomes a relatively ideal therapeutic target in AD (Hata et al., 2016; Kumar et al., 2017). Combined rTMS/PET studies have demonstrated that high frequency rTMS over the DLPFC increases cerebral blood flow in the DLPFC and anterior cingulate cortex (ACC; Paus et al., 2001), and modulates dopaminergic activity in the ACC and orbitofrontal cortex (Cho and Strafella, 2009), but as well as serotonergic activity in the cingulate, insular, and parahippocampal cortices (Sibon et al., 2007). Ahmed et al. (2012) compared the effects of low (1 Hz) versus high (20 Hz) frequencies of rTMS in AD and showed that the high frequency rTMS group improved significantly more in all rating scales and at all time points after treatment. Li et al. (2017) used a 10 Hz rTMS over the left DLPFC in young healthy participants and found improved performance on Stroop task and larger frontocentral N2 and N450 amplitudes. Hong et al. (2021) employed cerebral ischemic rats to demonstrate high frequency rTMS improves cognitive function by regulating synaptic plasticity. Thus it has been suggested that high-frequency rTMS over the left DLPFC could recruit more neural resources or enhance neural efficiency through electrophysiologically excitatory effect and cortical plasticity to contribute to better improvement (Li et al., 2017).

Second, considering the poor effect of pharmacotherapy in patients with severe AD, we adopted a longer rTMS course (60 days) with higher stimulus intensity (100% MT), which was different from most previous studies in mild and moderate AD. The excitability of the cerebral cortex depends on the intensity of TMS stimulation. Studies have revealed that TMS stimulation of the parietal lobe in healthy subjects with a 90% MT can improve motor cortex excitability. While for patients with AD, 110% of MT intensity is required. The long-term and short-term treatment effects have been compared in several meta studies. A previous meta-analysis of Lin et al. (2019) showed that long-term rTMS (more than 5 times) had a better effect on the improvement of cognitive function in patients with AD than short-term rTMS (less than 3 times). It has been demonstrated that long-term rTMS enhances LTP in AD patients (Li et al., 2021b). For pharmacological trials, a longer duration of 6–48 months is typically recommended for slowing progression of symptoms (Rajji, 2019). While the effect of long duration of rTMS treatment is lacking, our results demonstrated the feasibility and effectiveness of long-term therapy in moderate and severe AD.

Third, we predicted the effect of rTMS treatment for AD patients based on baseline resting-state FC. Notably, the multivariate FC of the right hippocampus and posterior parahippocampal gyrus from the medial temporal lobe (MTL) was found to be associated with individual difference in gains from the rTMS treatment. The MTL is the earliest and most severely damaged area of AD and plays a key role in the disease (Berron et al., 2020). Numerous studies have linked baseline and longitudinal hippocampal functional connectivity to cognitive changes in progression of MCI and AD (Wang et al., 2011; Dautricourt et al., 2021). Engvig et al. (2012) found that larger pre-training hippocampal volumes were positively associated with more memory improvements after a strategy-based cognitive training program in subjective memory complaints. Furthermore, Gallen et al. (2016) found that older adults with more segregated brain sub-networks at baseline exhibited greater training improvements in the ability to synthesize complex information. Vermeij et al. (2017) demonstrated that a ‘youth-like’ prefrontal activation pattern at older age is associated with more gains and cognitive plasticity from a working-memory training. Previous studies have also proposed that patients with better cognitive performance have less damage to brain functional connections and require less TMS stimulation (Kahkonen et al., 2005; Bonni et al., 2013). Here, our finding preliminarily proposed the possibility of resting-state multivariate FC as a pre-treatment neuroimaging marker for predicting treatment outcomes. It would be necessary in future large sample studies to further examine how the baseline functional connectivity particularly for the MTL areas contributes to individual differences in treatment outcomes, and if a higher baseline MTL functional connectivity is associated with more benefits from the rTMS treatment.

As a rehabilitation therapy for AD, rTMS has demonstrated potential in improving cognitive function and psychobehavioral symptoms in patients with moderate-to-severe AD. The limitation of this clinical research is that the sample size for neuroimaging studies was small and there was no long-term follow-up study to observe the duration of efficacy. Although we demonstrated the baseline FC as the potential biomarker of rTMS treatment outcomes in behavioral performance, using them as objective and quantitative neuroimaging biomarker for personalized evaluation of therapeutic effect before rTMS treatment still requires larger sample study to evaluate the stability and robustness of the connectivity features. In addition, the neural changes induced by the rTMS remains to be investigated in the future. To examine the neural reorganization pattern underlying rTMS treatment would provide more mechanic understanding of the rTMS in improving psychobehavioral symptoms of AD patients. Another focus of rTMS in the future should be on key problems, such as adopting multiple site stimulation, improving the clinical efficacy of rTMS by using MRI navigation, and other technologies for accurate positioning.

In conclusion, the present study aimed to evaluate the treatment efficacy of rTMS for moderate and severe AD patients, and the underlying neural contribution to individual difference in treatment outcomes. Hence, we performed a randomized, sham-controlled rTMS trail with a 60-session long, 10 Hz stimulation of the left DLPFC. The results showed that the rTMS treatment significantly improved cognitive performance, reduced psychiatric symptoms, and improved the clinician’s global impression of change of these patients. Furthermore, our finding preliminarily proposed the possibility of resting-state multivariate FC in (para) hippocampal region and frontal and occipital clusters as a pre-treatment neuroimaging marker for predicting treatment outcomes.

## Data availability statement

The raw data supporting the conclusions of this article will be made available by the authors, without undue reservation.

## Ethics statement

The studies involving human participants were reviewed and approved by Beijing Geriatric Hospital (BGH) Ethics Committee. The patients/participants provided their written informed consent to participate in this study.

## Author contributions

SZ and RL designed this study. LL, LZ, LM, HW, MC, and XH recruited participants and collected their information. RL, LL, and LZ performed data analysis, data management, and reference collection. SZ and RL wrote the manuscript. All authors contributed to the article and approved the submitted version.

## Funding

This study was supported by the National Natural Science Foundation of China (grant no. 62177004).

## Conflict of interest

The authors declare that the research was conducted in the absence of any commercial or financial relationships that could be construed as a potential conflict of interest.

## Publisher’s note

All claims expressed in this article are solely those of the authors and do not necessarily represent those of their affiliated organizations, or those of the publisher, the editors and the reviewers. Any product that may be evaluated in this article, or claim that may be made by its manufacturer, is not guaranteed or endorsed by the publisher.

## Footnotes

^1^ https://web.conn-toolbox.org

^2^ https://neuro-jena.github.io/cat/
